# Low-Concentrations of Fatty Acids Induce an Early Increase in IL-8 Levels in Normal Human Astrocytes

**DOI:** 10.3390/metabo12040329

**Published:** 2022-04-06

**Authors:** Ana-Maria Dobri, Elena Codrici, Ionela-Daniela Popescu, Lucian Albulescu, Emanuel Tudor Fertig, Ana-Maria Enciu, Cristiana Tanase, Mihail E. Hinescu

**Affiliations:** 1 “Victor Babes” National Institute of Pathology, 050096 Bucharest, Romania; ana-maria.dobri@drd.umfcd.ro (A.-M.D.); elena.codrici@ivb.ro (E.C.); daniela.popescu@ivb.ro (I.-D.P.); lucian.albulescu@ivb.ro (L.A.); emanuel.fertig@ivb.ro (E.T.F.); bioch@vbabes.ro (C.T.); mhinescu@ivb.ro (M.E.H.); 2 Faculty of Medicine, “Carol Davila” University of Medicine and Pharmacy, 050474 Bucharest, Romania; 3 Faculty of Medicine, “Titu Maiorescu” University, 031593 Bucharest, Romania

**Keywords:** fatty acids (FA), palmitic acid (PA), oleic acid (OA), pro-inflammation cytokines, IL-6, IL-8

## Abstract

Fatty acids (FAs) have been shown to exhibit a pro-inflammatory response in various cell types, but astrocytes have been mostly overlooked. FAs, both saturated and unsaturated, have previously been shown to induce pro-inflammatory responses in astrocytes at high concentrations of hundreds of µg/mL. SSO (Sulfo-*N*-succinimidyl Oleate sodium), an inhibitor of FA translocase CD36, has been shown to prevent inflammation in the mouse brain by acting on local microglia and infiltrating monocytes. Our hypothesis was that SSO treatment would also impact astrocyte pro-inflammatory response to FA. In order to verify our assumption, we evaluated the expression of pro- and anti-inflammatory cytokines in normal human astrocyte cell culture pre-treated (or not) with SSO, and then exposed to low concentrations of both saturated (palmitic acid) and unsaturated (oleic acid) FAs. As a positive control for astrocyte inflammation, we used fibrillary amyloid. Neither Aβ 1–42 nor FAs induced CD36 protein expression in human astrocytes in cell culture At low concentrations, both types of FAs induced IL-8 protein secretion, and this effect was specifically inhibited by SSO pre-treatment. In conclusion, low concentrations of oleic acid are able to induce an early increase in IL-8 expression in normal human astrocytes, which is specifically downregulated by SSO.

## 1. Introduction

Long-chain fatty acids (LCFA), both saturated and unsaturated, in addition to their structural and energetic roles, can actively modify cellular behaviour by binding to several transcription factors, such as peroxisome proliferation activating receptors (PPARs) or NF-kB [1,2,3]. Both pathways are related to inflammation [4,5,6], and many non-immune cell types respond to FA treatments by producing cytokines, such as skin cells [7,8], intestinal epithelial cells [9], hepatic cells [10], and muscle cells [11]. FA are translocated into cells via CD-36, a transmembrane protein widely expressed on various cell types. This receptor also acts as a scavenger receptor, binding, amongst others, hydrophobic peptides such as amyloid peptide 1–42, involved in Alzheimer’s disease (AD) [12,13,14].

Astrocytes are also able to respond to FA treatment by producing cytokines, albeit they do not express CD36 in basal conditions. However, data that support this mostly focus on saturated FA. High concentrations of saturated FA (1000 µM or higher), such as palmitic acid (PA), have been reported to induce synthesis of pro-inflammatory cytokines (Il-6 and TNFalfa) in astrocyte cell cultures in a dose-dependent manner [15,16,17]. Furthermore, PA was proposed as “an intracellular signalling molecule involved in disease development”, as it induces (among other mechanisms) inflammation and subsequent neurodegeneration (reviewed in [18]). Conversely, unsaturated FA, such as oleic acid (OA), have been reported not to induce the release of pro-inflammatory cytokines, regardless of the tested dose [19].

SSO, known as an inhibitor of FA uptake, was previously shown to inhibit inflammation in the mouse brain by acting on local microglia and infiltrating monocytes [20]. However, as the majority of glial cells are represented by astrocytes, the impact of SSO on astrocyte behaviour is of interest. The aim of this study was to determine whether SSO treatment would impact pro-inflammatory cytokine production in astrocytes.

## 2. Results

### 2.1. Astrocytes Are Able to Uptake Both Saturated and Unsaturated Fatty Acids, Which Are Not Impaired by Pre-Treatment with SSO

**Figure 1:**
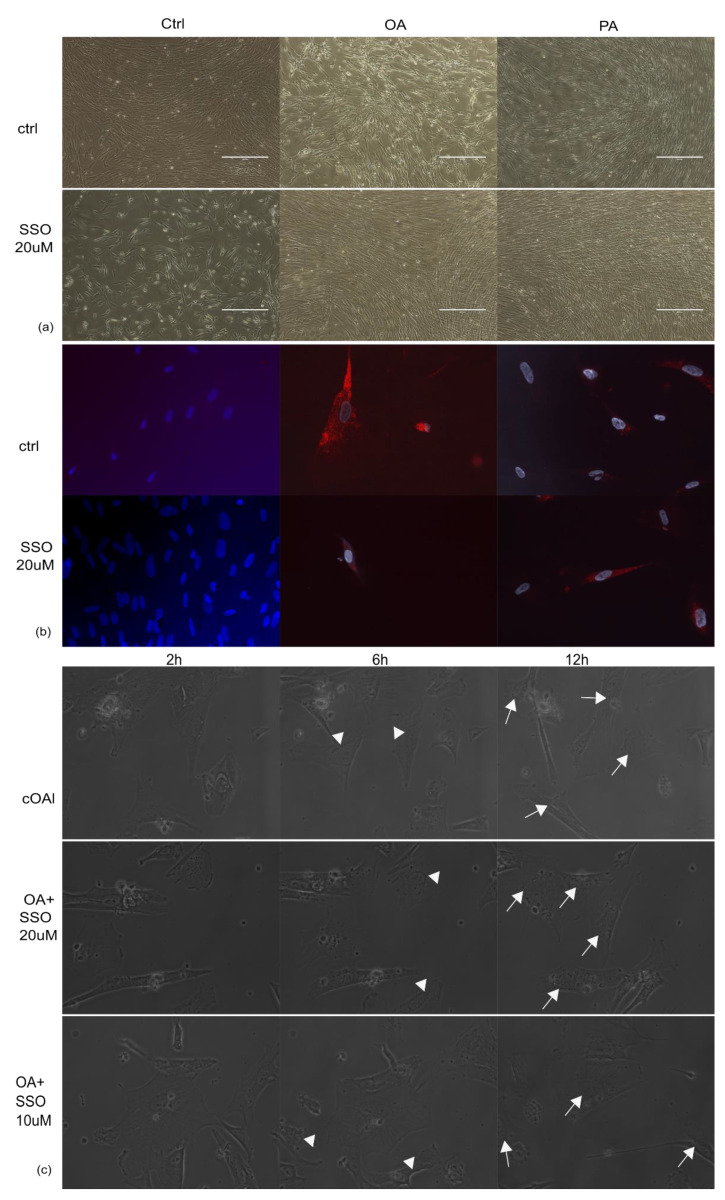
(**a**) Addition of low concentrations of either saturated FA (palmitic acid (20 µM) or unsaturated FA (oleic acid, 40 µM) rescue normal human astrocyte proliferation in cell culture. Phase-contrast, 10×. Assessment of lipid droplet formation in the presence of SSO pre-treatment by OilRed O stain (**b**) and video microscopy (**c**). SSO treatment does not impede lipid granule formation in the presence of OA, nor the timeline of their accumulation inside cells. In SSO-treated, as well as non-treated cells, granules are visible inside astrocytes as soon as 6 h (arrow heads) and clearly observed at 12 h (arrows).

First, we addressed the question of whether SSO would affect human astrocytes in cell culture. Because SSO is a known inhibitor of FA uptake, we also added a non-saturated FA (oleic acid, OA) or a saturated fatty acid (palmitic acid, PA) in addition to SSO. We observed that while SSO impairs cell proliferation, both the saturated and unsaturated FA rescued this effect (Figure 1a). Dosage of SSO and time of pre-incubation was chosen based on previous studies [21,22] and on the observation that times longer than 15 min resulted in cell detachment.

Next, we tested whether astrocytes are indeed able to uptake both types of FAs from the cell culture medium and whether pre-treatment with SSO blocks their uptake. Because OilRed Stain indicated the presence of lipid inclusions regardless of SSO treatment (Figure 1b), we also analysed dynamic lipid droplet formation under OA treatment, in the presence or absence of SSO pre-treatment, using video microscopy (Figure 1c).

### 2.2. In the Presence of OA, SSO Short-Term Treatment Does Not Impair Long-Term Astrocytes Viability, as Assessed by Video Microscopy

**Figure 2:**
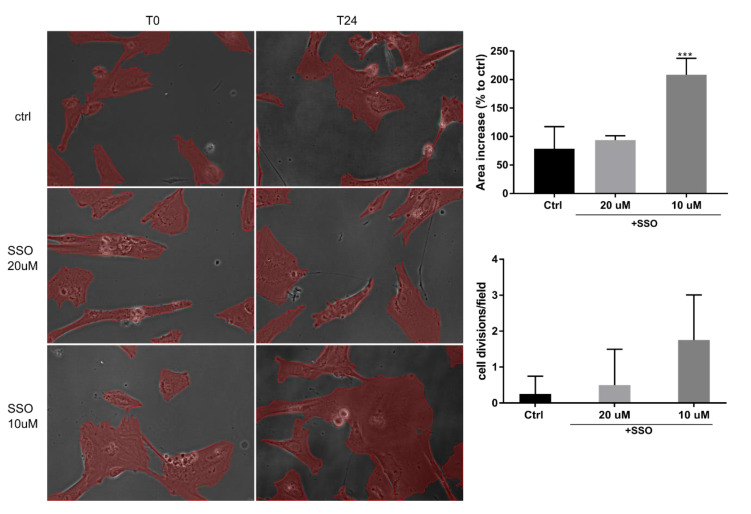
Short-term treatment of NHAs with SSO does not affect their viability or cell division rate. NHAs were pre-treated with SSO for 10 min at indicated concentrations, then incubated for 24 h in a time-lapse incubator. The covered area was measured post-hoc at indicated times using NisElements Br. Four fields were measured for each situation, and the statistical analysis was performed using one- way ANOVA, Dunnett multiple comparison, where data were compared to control (*** *p* < 0.001).

Next, we were interested in confirming in real-time whether short-term treatment (10 min) with SSO in two different concentrations (10 and 20 μM) affects the viability of normal human astrocytes (NHA) over 24 h. We were also interested in quantifying the spread (covered area) of NHAs (equivalent, for example, with scar formation) and whether the modification in the spread is associated with modified cell proliferation. Following SSO removal, cell medium was replaced with OA 40 μM for all tested situations. There was no noticeable difference in cell mobility or viability between the tested conditions. However, we noticed that a lower SSO concentration led to a higher area coverage, possibly related to a slightly higher number of cell divisions (Figure 2).

### 2.3. Astrocytes Are Able to Produce Pro-Inflammatory Cytokines in Cell Culture, and Their Synthesis Is Lowered by SSO Treatment

**Figure 3:**
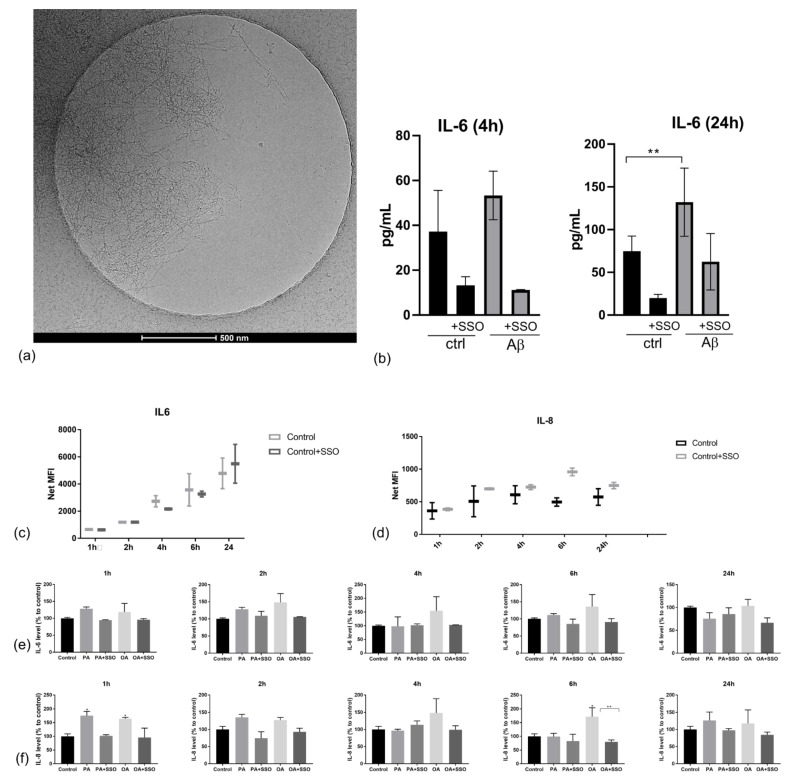
Assessment of cytokine release in NHA culture. NHAs were incubated with fibrillary 1–42β-amyloid 0.2 μM (as verified by cryo-EM (**a**) and IL-6 was measured by ELISA from cell culture medium at indicated times (**b**). The dynamics of IL-6 and IL-8 levels, w/o SSO pre-treatment (20 μM, 10 min) were also measured in the cell culture medium using multiplexing (**c**,**d**). Levels of IL-6 (**e**) and IL-8 (**f**) in the presence of PA or OA in cells pre-treated with SSO were evaluated by xMAP array. Data are reported as a percentage to control. Statistical analysis was performed by one-way ANOVA, Dunnett multiple comparison, where data were compared to control (* *p* < 0.05, ** *p* < 0.01).

To confirm that NHAs are able to produce a pro-inflammatory response in cell culture, we treated them with 0.2 µM β amyloid 1–42, preincubated for 48 h at 37 °C for fibril formation. At the selected concentration, the formation of fibrils was documented by cryo-EM as early as 24 h (Figure 3a). IL-6 was determined from the cell supernatant at 4 and 24 h. As expected, β amyloid 1–42 treatment induced IL-6 formation at 24 h, which was downregulated by SSO treatment (Figure 3b). Additionally, SSO treatment had a lowering effect on cytokine production on all tested conditions, which prompted us to investigate further the production of other pro-inflammatory cytokines in cell culture. Of all tested pro- and anti-inflammatory cytokines (IL-1b, IL-4, IL-6, IL-8, IL-10, TNFa, MIP1a), only IL-6 and IL-8 were detected in NHA cell culture supernatants, but were secreted at different times. Over 24 h, IL-6 registered continuous synthesis (Figure 3c), whereas IL-8 reached a peak at 6 h (Figure 3d). Furthermore, OA induced a significant increase in IL-8 levels after 6 h of treatment. Finally, SSO pre-treatment induced normalization of IL-6 (Figure 3e) and IL-8 levels (Figure 3f).

## 3. Discussion

Even though neuroinflammation is associated with microglia activation, astrocytes have also the ability to secrete various cytokines in cell culture even in basal conditions, including IL-6 and IL-8 [23]. The pro-inflammatory activity of astrocytes can be induced to high levels by IL-1β and TNFα [24,25] and viral infections, e.g., HIV [26], to facilitate an immune response.

Astrocytes also contribute to the sterile inflammation of the central nervous system, associated with neurodegenerative diseases and activation of microglia on centre-stage [27,28]. Astrocytes have been demonstrated to react to Aβ 1–42, a hydrophobic peptide produced in Alzheimer’s disease (AD), by activating the Nf-kB signaling pathway [29], a known inducer of gene expression for pro-inflammatory cytokines.

Saturated FAs, such as PA, are non-immune inductors of sterile inflammation. In addition to its pro-inflammatory effect, PA was previously reported to be cytotoxic for astrocytes in concentrations of 100 µM or higher [17,30]. Using both Aβ 1–42 and PA at low concentration, we were able to detect an increase in IL-6 synthesis, by both ELISA and multiplexing.

Although astrocytes are not known to express CD36 in basal conditions, we asked the question as to whether it might be upregulated by Aβ 1–42 or FA (saturated or unsaturated) treatments, and whether the subsequent inflammation could be downregulated by SSO treatment, a known inhibitor of CD36. Dhungana et al. explored the effect of in vivo SSO treatment on a rodent model of stroke [20], referencing two other articles which administered SSO in animals [31,32], raising the possibility of SSO as a potential drug. In their study, the effect on astrocytes has not been documented. Although in our experimental setup the expression of CD36 was not up-regulated in the presence of neither Aβ 1-42 or FA, the SSO treatment did have an impact on cytokine production, on both IL-6 (when induced at significant levels with Aβ 1–42) and IL-8.

IL-8 increase was induced by OA during the first 6 h of treatment, which, to our knowledge, has not been previously reported. OA is known to induce PPAR-dependent gene transcription in yeast and murine cells [33,34], and astrocytes are known to express PPARα [35]. However, the focus is mostly on OA interaction with PPARγ, correlated to its anti-inflammatory effect [36], due to the ability of PPARγ to block Nf-KB binding to consensus gene sequences [35]. The correlation between OA, PPARα, and IL-8 is yet to be investigated. It is known that IL-8 is a chemoattractant for microglia and neutrophils (especially in central nervous system disorders with a leaky blood-brain barrier) and exerts an autocrine effect, which leads to neuroinflammation with deleterious effects on neurons. Most studies focus on microglia (an interesting mini-review [37] summarizes findings from human samples, cerebrospinal fluid sampling, and in vitro models of microglial IL-8 activation). Several studies showed that astrocytes are also able to produce IL-8 in response to various stimuli: 1 methyl 4 phenyl pyridine (MPP+, used to induce Parkinson’s disease in murine models) [38], Aβ 1–42 (a hydrophobic peptide which accumulates in Alzheimer’s disease) [29], cadmium chloride [39], even high glucose [40]. Our study adds to this list OA in low concentration. IL-8 can exert neuronal effects via its receptors, shown to be expressed by a variety of neurons (glutamatergic, gamma–aminobutyric acidergic, and cholinergic neurons [41]). Also, it has been shown that increased IL-8 can be detrimental to neurons (retinal ganglion cells [42]; cholinergic septal neurons [43]) and it has been proposed that pharmacological inhibition of IL-8 receptors can prove beneficial in AD ([44]). According to Bylicky et al. [45]) there are at least seven mechanisms by which astrocytes exert neuroprotective roles, and their role in modulation and regulation of immune responses and neuroinflammation is also discussed, but not specifically related to IL-8. The specific role of IL-8 in orchestration of these events is still to be determined.

Although classically reported as an inhibitor of CD36-mediated translocation of FA [21], a recent study showed that SSO might act on intracellular proteins, rather than transmembrane transporters [46], which would explain why astrocytes are able to respond to SSO treatment, despite the lack of the canonical target of SSO: the transmembrane CD36 FA translocase. Similar to Dhungana et al. [20], this study also showed that SSO could be useful to mitigate neuroinflammation but with a direct effect on astrocytes. As for translatability to human studies, there next step would be testing of SSO on brain organoids. To our knowledge, there are no such studies so far.

## 4. Materials and Methods

### 4.1. Cell Culture and Cell Treatments

Normal human astrocytes (NHA, Lonza) were routinely grown in ABM Basal Medium (CC-3187) supplemented with AGM SingleQuotsTM Supplements (CC-4123) in a cell culture incubator (5% CO_2_, 37 °C). For subsequent treatments and sampling, 5000 cells/cm^2^ were seeded for 1 week in 6-well plates, with periodic medium change.

Beta-amyloid peptide (1–42) (ab 120301, Abcam) and the corresponding inactive control (ab120481) were reconstituted to a 200 µM stock in NaOH 10 mM/HEPES 25 mM, diluted to working concentration (0.2 µM), and incubated in complete cell medium up to 48 h in a cell culture incubator (5% CO_2_, 37 °C), for fibril formation.

Sulfo-*N*-succinimidyl Oleate sodium (SSO) (Sigma, SML2148) was reconstituted in DMSO to 5.2 mM stock concentration and diluted to working concentration (20 µM) with complete cell medium. Control cells were incubated with vehicle (complete cell medium supplemented with the respective concentration of DMSO 1/200). Cells were pre-treated with a working concentration of SSO for 10 min, then the medium was replaced with normal cell medium, with or without FAs (PA and OA).

PA (Sigma P0500) and OA (Sigma O1008) were reconstituted in 70% ethanol to a 40 mM stock concentration, which was diluted to working concentrations (OA 40 µM and PA 20 µM) with complete cell medium. Corresponding controls were incubated with vehicle (medium supplemented with 0.07% ethanol).

### 4.2. Videomicroscopy

A total of 10,000 cells were incubated in 4 chamber dishes (80416, Ibidi), left to adhere overnight, and pre-treated for 10 min with two concentrations of SSO (20 and 10 µM). After treatment, SSO medium was replaced with vehicle or OA 40 µM. Images were captured in a Nikon Biostation every 15 min, and the nude area was assessed with NiS Elements BR software.

OilRed O staining: Cells were fixed in 3.7% formaldehyde for 30 min to 1 h, washed with water, and then incubated with 60% isopropanol for 5 min. Oil Red O Stock solution (Sigma-Aldrich) was reconstituted with 100% isopropanol. Oil Red O Working solution was prepared 15 min before staining by mixing 3 parts of Oil Red O Stock solution and 2 parts of water, and then the solution was filtered through Whatman No.1 filter paper. The cells were stained with Oil Red O Working solution for 10 min at room temperature and afterwards washed 2–5 times with water until no excess stain was seen. DAPI staining was performed after this step for 10 min at room temperature, and protected from light. The red lipid droplets were visualised by using fluorescent microscopy with a Nikon TI inverted fluorescence microscope.

### 4.3. Electron Cryomicroscopy

Stock solutions of beta-amyloid (1–42) and inactive control were resuspended in HEPES 25 mM. A 5 µL drop of each sample was deposited on glow-discharged copper grids (Quantifoil R2/2, Quantifoil Micro Tools), then frozen by rapid immersion in liquid ethane using a Leica EM-GP (Leica Microsystems). Grids were then transferred under liquid nitrogen to a Gatan 626 cryo-holder (Gatan) and examined on a Talos 200C S/TEM (Thermo Scientific, Coon Rapids, MN, USA), equipped with a 4 k × 4 k Ceta camera. Images were acquired using low dose settings, at a nominal magnification of 36,000× and at 3 to 5 µm under focus, giving a final object sampling of 4.1 Å per pixel.

### 4.4. ELISA

IL-6 was measured using commercial Legend Max quantitative assays (BioLegend, San Diego, CA, USA). Standards and samples: cell culture supernatants (50 μL) were added to the plates and incubated at RT for 2 h, with shaking at 200 rpm. All further incubations with detection antibody (100 μL), Avidin-HRP solution (100 μL), Substrate Solution (100 μL), and Stop Solutions (100 μL) were performed according to manufacturer protocol. Samples were analysed in duplicate, and the absorbance was read at 450 nm and 570 nm, using a Sunrise-Basic Tecan Microplate Reader (Tecan Group Ltd., Männedorf, Switzerland). The absorbance at 570 nm was subtracted from the absorbance at 450 nm. The data were calculated with computer-based curve-fitting software (Magellan) using a 5-parameter logistics curve-fitting algorithm. IL-6 was expressed in picograms per millilitre of the sample.

### 4.5. Multiplexing

Cell culture supernatants were obtained as mentioned above, and then a Multiplex Magnetic Luminex Assay Human Premixed Multi-Analyte analysis (R&D Systems, Minneapolis, MN, USA) was performed to assess the levels of 7 different biomarkers (IL-1 beta, IL-6, IL-8, IL-10, IL-12p70, TNFa, VEGF-A). Briefly, the magnetic beads were incubated with buffer, cytokine standards, or samples, according to manufacturer protocols. All further incubations with Biotin–Antibody cocktail and Streptavidin–Phycoerythrin were performed at room temperature, in the dark, with shaking at 800 ± 50 rpm. Multiplex data acquisition was achieved using the Luminex^®^200^TM^ platform (Luminex Corp, Austin, TX 78727, USA), and analysis was performed using xPONENT 4.2 software; the calibration curves were generated with a 5-parameter logistic fit. Duplicate samples were used for all specimens, and the average concentrations were used for statistical analysis.

### 4.6. Statistical Analysis

Statistical data analysis was performed with GraphPad v7, by one-way ANOVA, Dunnett multiple comparison, where data were compared to control (* *p* < 0.05, ** *p* < 0.01, *** *p* < 0.001, **** *p* < 0.0001).

## 5. Conclusions

Astrocytes are active participants in the pro-inflammatory milieu in sterile inflammation, induced, for example, by fibrillar amyloid or saturated FA, such as PA. At high concentrations, saturated FAs are known to induce an increased expression of pro-inflammatory cytokines (IL-6, TNFα) in astrocytes. Conversely, monosaturated FA, such as OA, had a protective effect. Here, we provide evidence that while SSO does not interfere with the ability of astrocytes to uptake FAs, it significantly interferes with pro-inflammatory cytokine release in the presence of both pathological stimuli (fibrillary 1–42 beta-amyloid) as well as low concentrations of FAs. In addition, we found that OA at low concentration stimulates the early release of IL-8 by a mechanism needing to be further determined.

## Data Availability

The raw data presented in this study are available on request from the corresponding author, because of its usage in the ongoing study.
